# Direct, Acute Effects of Klotho and FGF23 on Vascular Smooth Muscle and Endothelium

**DOI:** 10.1371/journal.pone.0093423

**Published:** 2014-04-02

**Authors:** Isabelle Six, Hirokazu Okazaki, Priscilla Gross, Joanna Cagnard, Cédric Boudot, Julien Maizel, Tilman B. Drueke, Ziad A. Massy

**Affiliations:** 1 INSERM Unit 1088, Jules Verne University of Picardie, Amiens, France; 2 Amiens University Medical Center, Amiens, France; Max-Delbrück Center for Molecular Medicine (MDC), Germany

## Abstract

Chronic kidney disease (CKD) is regarded as a state of Klotho deficiency and FGF23 excess. In patients with CKD a strong association has been found between increased serum FGF23 and mortality risk, possibly via enhanced atherosclerosis, vascular stiffness, and vascular calcification. The aim of this study was to examine the hypothesis that soluble Klotho and FGF23 exert direct, rapid effects on the vessel wall. We used three in vitro models: mouse aorta rings, human umbilical vein endothelial cells, and human vascular smooth muscle cells (HVSMC). Increasing medium concentrations of soluble Klotho and FGF23 both stimulated aorta contractions and increased ROS production in HVSMC. Klotho partially reverted FGF23 induced vasoconstriction, induced relaxation on phosphate preconstricted aorta and enhanced endothelial NO production in HUVEC. Thus Klotho increased both ROS production in HVSMC and NO production in endothelium. FGF23 induced contraction in phosphate preconstricted vessels and increased ROS production. Phosphate, Klotho and FGF23 together induced no change in vascular tone despite increased ROS production. Moreover, the three compounds combined inhibited relaxation despite increased NO production, probably owing to the concomitant increase in ROS production. In conclusion, although phosphate, soluble Klotho and FGF23 separately stimulate aorta contraction, Klotho mitigates the effects of phosphate and FGF23 on contractility via increased NO production, thereby protecting the vessel to some extent against potentially noxious effects of high phosphate or FGF23 concentrations. This novel observation is in line with the theory that Klotho deficiency is deleterious whereas Klotho sufficiency is protective against the negative effects of phosphate and FGF23 which are additive.

## Introduction

Cardiovascular disease (CVD) is the leading cause of mortality and morbidity in patients with chronic kidney disease (CKD) [1]. Endothelial dysfunction occurs since the very early stages of CKD [2], and is regarded as an important contributor to increased CVD risk [3]. Endothelial dysfunction is a systemic pathological condition which can be defined as resulting from an imbalance between the actions of vasorelaxing and vasoconstrictor factors. The imbalance is mainly caused by reduced nitric oxide (NO) bioavailability and/or increased generation of reactive oxygen species (ROS) [4].

CKD is considered as a state of Klotho deficiency. α-Klotho (Klotho), originally identified as an anti-aging gene, is predominantly expressed in the kidney but is also detectable in other organs such as parathyroids, choroid plexus and human vascular tissue [5]–[7]. As a co-factor of fibroblast growth factor 23 (FGF23), Klotho is involved in the control of renal phosphate handling and 1,25 diOH vitamin D synthesis [8]–[10]. Klotho-deficient and FGF23-deficient mice exhibit similar phenotypes, characterized by accelerated aging, atherosclerosis, ectopic calcifications, bone demineralization, skin atrophy and emphysema [11], [12].

Klotho deficiency leads to FGF23 resistance since Klotho acts as an obligatory co-receptor in various tissues probably including the vessel wall [7], [13], [14]. However, high circulating FGF23 levels can also exert Klotho independent deleterious CV effects [15]. Excessive FGF23 levels were found to be associated with impaired vasodilatation [16], and Klotho gene deficiency to interfere with endothelium-dependent vasodilatation [17]. Klotho gene delivery improves vascular function through increased endothelial-derived NO production and less oxidative stress in VSMCs [18].

Collectively, ample evidence suggests that Klotho and FGF23 play a role in vascular function. However, the interaction between Klotho and FGF23 has recently been found to be more complex than previously thought, requiring further clarification. In the present study, we aimed to determine direct, rapid effects of Klotho and FGF23 on vascular function and to investigate potentially underlying mechanisms. We first examined the vascular reactivity of mouse aorta to exogenous soluble Klotho and FGF23 and observed direct effects. We next asked whether the effects were related to abnormal NO and ROS generation, and then investigated potentially involved signaling pathways using human umbilical vein endothelial cells (HUVEC) and human vascular smooth muscle cells (HVSMC).

## Materials and Methods

### Ethics Statement

HVSMC were isolated in our laboratory using an explant technique from aortic tissue of healthy donors. Samples were obtained after aortic valve bypass surgery or other types of surgery on the aorta from patients with various cardiovascular diseases (Pr Caus, Pôle Coeur Thorax Vaisseaux, CHU Amiens, France) who gave informed written consent in accordance with French legislation, under PROTOCOL N°2009/19. This protocol was approved by the Ethics Committee, CPP Nord-Ouest II. The investigations were performed according to the principles outlined in the Declaration of Helsinki for use of human tissue or subjects. All of the animal studies were conformed to the principles of European Commission guidelines and all protocols were approved by our Institution's Animal Care and Use Committee (CREMEAP, protocol N° 2006/B7).

### Products

For the experiments, we used Recombinant Human soluble form of Klotho and Recombinant intact Human FGF23 (R&D Systems, Minneapolis, USA). Endothelial Cell Growth kit-BBE was obtained from ATCC (Manassas, VA, USA). Phosphate (NaH_2_PO_4_), Dulbecco's Modified Eagle's Medium (DMEM) and penicillin/streptomycin (P/S) were purchased from Sigma Aldrich (Saint Louis, USA). GlutaMAX™ (Glut) and Fluorescent probe, 2′, 7′dichlorodihydrofluorescein diacetate (DCF) were obtained from Invitrogen, (Saint Aubin, France) and fetal calf serum (FCS) from Dominique Dutcher Laboratories (Brumath, France). FACS Canto II flow cytometer and FACSDiva software were obtained from BD Biosciences (Rungis, France). 4-amino-5-methylamino-2′, 7′-difluorofluorescein diacetate (DAF-FM DA) was obtained from Merck Millipore (St. Quentin-en-Yvelines, France).

All the primary antibodies for western blot analyses were obtained from Cell Signaling, Ozyme (St. Quentin en Yvelines, France).

### Animals and diet

All animal studies were performed using C57/BL-6 wild-type female mice at age 8 weeks. The mice were purchased from Charles Rivers Laboratories (Lyon, France). They were housed in polycarbonate cages in temperature- and humidity-controlled rooms with a 12-h light/dark cycle and given free access to water and regular laboratory chow (Diet 2918, Harlan, Oxon, UK).

Mice were anesthetized by intraperitoneal injection of pentobarbital sodium (150 mg/kg) and at the experimental endpoint euthanasia was accomplished by removal of the heart.

### Ex vivo models

#### Vasoreactivity experiments

Wild-type mice were anesthetized and a midline incision was made through the sternum to open up the thoracic cavity, and the descending thoracic aorta was carefully isolated. Each aorta was sectioned into 3.5 mm rings devoid of fat and connective tissue. The rings were placed in Kreb's-Henseleit (KH) solution under 5% CO_2_ and 95% O_2_ atmosphere at 37*°C*. The aorta rings were maintained under a 1.3 g tension (previously determined as the optimal point of their length-tension relationship) and allowed to equilibrate for 60 min. The aorta rings were constricted with KCl (70 mM) and then washed out to reach the resting level.

For the study of potential vasoconstrictor effects of phosphate, Klotho or FGF23, aorta rings were exposed to cumulative treatments with phosphate (1–3 mM), recombinant mouse Klotho (0–2 nM) or recombinant mouse FGF23 (0–400 ng/ml). As the vessels were not preconstricted, only vasoconstriction was observed. To test the implication of oxygen-derived free radicals or ERK, we incubated vessels in the presence of dimethylthiourea (10 mM), a scavenger of hydroxyl radical or the ERK inhibitor U0126 (10 μM) for 40 min.

The vascular effect of Klotho in association with FGF23 was studied by exposing aorta rings to both Klotho (0.8–1.6 nM) and FGF23 (10–200 ng/ml). Vascular wall tension was supplied by a force-displacement transducer and changes in isometric force recorded continuously. The contractile response was expressed as percentage of KCl contraction, taking into account resting contraction level.

The vascular effect of phosphate in association with Klotho was studied by exposing vessels to phosphate 2.0 mM followed by a range of Klotho concentrations (0.4–2.0 nM). Contraction values were expressed as percentage of contraction obtained with KCl, taking into account resting contraction level. The relaxation obtained with Klotho was calculated taking into account the maximal contraction obtained with phosphate 2.0 mM. A possible involvement of NO was studied by incubating vessels with L-NNA (a competitive inhibitor of nitric oxide synthase, with selectivity for the neuronal and endothelial isoforms of the enzyme) for 30 min before stimulation with phosphate and Klotho. The role of endothelial cells in arterial relaxation was studied by using aortic rings devoid of endothelium. The endothelium was removed by gently rubbing off the intimal surface of the aortic rings with a wooden stick. The effectiveness of this removal was demonstrated by the lack of response to acetylcholine (up to 1.0 μM).

The vascular effect of phosphate in association with FGF23 was studied by stimulating vessels with phosphate 2.0 mM, followed by a range of FGF23 concentrations (10–200 ng/ml). Contraction values were expressed as percentage of contraction obtained with KCl, taking into account resting contraction level.

The vasoconstrictor effects of phosphate and Klotho in association with FGF23 were studied by exposing aortic rings to phosphate (2.0 mM), Klotho (1.6 nM) and FGF23 (10 ng/ml) together.

The effects of these 3 drugs were confirmed by concomitant vessel incubation with phosphate (2 mM), Klotho (1.6 nM) and FGF23 (10 ng/ml) followed by stimulation with phenylephrine (3.10^−5^ M). The contractile response was expressed as percentage of KCl contraction, taking into account resting contraction level.

The relaxation effects of phosphate (2.0 mM), Klotho (1.6 nM) and FGF23 (10 ng/ml) were studied by incubating vessels with these 3 drugs followed by stimulation with acetylcholine (3.10^−5^ M). Relaxation was calculated taking into account the maximal contraction obtained with phenylephrine.

### Western blot analysis of mouse aortas

The descending aorta was extracted from mice and placed into DMEM 1%, Glut 1%, P/S, 0% FBS. After stimulation with the respective reagents for 30 min, aortas were lysed. Protein concentration was determined by Bradford assay. Proteins were separated in 10% SDS–polyacrylamide gel electrophoresis and transferred to nitrocellulose membranes. The blots were then incubated overnight at 4°C with the following primary antibodies: anti-ERK1/2 (1∶1000) and anti-p-ERK1/2 (1∶1000). The blots were then incubated with secondary antibody to detect immuno reactive bands. The membranes were developed with the enhanced chemiluminescence detection system and a specific, peroxidase-conjugated anti-IgG antibody. Band intensity was analyzed using the GeneGenius Bio Imaging System and protein levels were normalized against total ERK1/2 for p-ERK1/2.

### In vitro models

#### Human umbilical vein cells (HUVEC)

HUVEC, obtained from ATCC, were cultured on 0.1% gelatin-coated flask in a specific culture medium (Vascular Cell Basal Medium) with endothelial Cell Growth kit-BBE.

#### Human vascular smooth muscle cells (HVSMC)

HVSMC were maintained in DMEM with 1% Glut, P/S and supplemented with 15% FCS.

The two cell types were maintained in culture at 37°C (5% CO2, 90% humidity) and used from passage 3 to passage 8.

### Incubation of HUVEC or HVSMC with different drugs

After overnight starvation in DMEM containing P/S, 1% Glut, 0.2% Bovine Serum Albumin (BSA) and 0% FCS, cells were incubated for 10 min at 37°C with DMEM 1% P/S, 1% Glut, containing phosphate (2.0 mM) and/or Recombinant Human Klotho (1.6 nM) and/or Recombinant Human FGF23 (10 ng/mL).

### Measurement of intracellular reactive oxygen species (ROS) production by HUVEC or HVSMC using flow cytometry

To evaluate ROS production, hydrogen peroxide (H_2_O_2_) levels were monitored using fluorescent probe, DCF. After overnight serum starvation, confluent cells were incubated with the different drugs. H_2_O_2_ production was measured by flow cytometry on detached cells resuspended in PBS and analyzed in a FACS Canto II flow cytometer. The analysis was focused on the whole cell population. Mean fluorescence intensity was calculated using FACSDiva software and expressed as percentage of control value.

### Measurement of intracellular NO production by HUVEC using flow-cytometry

NO production by HUVEC was assessed by measuring the fluorescence of DAF-FM DA, a specific NO probe. After overnight serum starvation, HUVEC were loaded with DAF-FM DA (1.0 μM) for 30 min. Subsequently, cells were stimulated with the different drugs for 10 min. The stimulated cells were then trypsinized and using flow-cytometry (FACSCantoII), a population of 10,000 cells was gated and segregated based on its relative fluorescence intensity. Analyses were done using FACSDiva software.

### Western blot analysis

After overnight incubation in starvation medium, HUVECs were stimulated with respective drugs for 10 min. After cell lysis, protein levels were quantified with Bio-Rad Dc Protein Assay kit. Proteins were separated in 10% SDS–polyacrylamide gel electrophoresis and transferred to nitrocellulose membranes. The blots were then incubated overnight at 4°C with the following primary antibodies: anti eNOS (1∶1000), anti iNOS (1∶1000), and anti phospho-eNOS (serine 1177 or threonine 495, 1∶1000). The membranes were developed with the enhanced chemiluminescence detection system and a specific, peroxidase-conjugated anti-IgG antibody. Band intensity was analyzed using the GeneGenius Bio Imaging System and protein levels were normalized against eNOS for phospho-eNOS and β-actin for iNOS.

### Statistical analysis

Results were expressed as means ± SEM. Differences between groups were evaluated using analysis of variance (ANOVA) to assess differences between individual mean values. Comparisons between more than two means were performed by using analysis of variance (ANOVA) with or without repeated measurements. If a significant difference was found, Scheffe's test for multiple comparisons was used to identify inter-group differences. For ROS and NO determinations and for western blotting, data were analyzed using a non parametric test (Kruskal-Wallis) to determine significant differences (p<0.05) in mean values between groups followed by Mann-Whitney *U* test to evaluate the significance of differences between groups. Differences were considered to be significant when p<0.05.

## Results

### Concentration-dependent vasoconstriction, stimulation of ROS concentration and activation of ERK pathway by phosphate, Klotho, and FGF23 respectively

**Figure 1:**
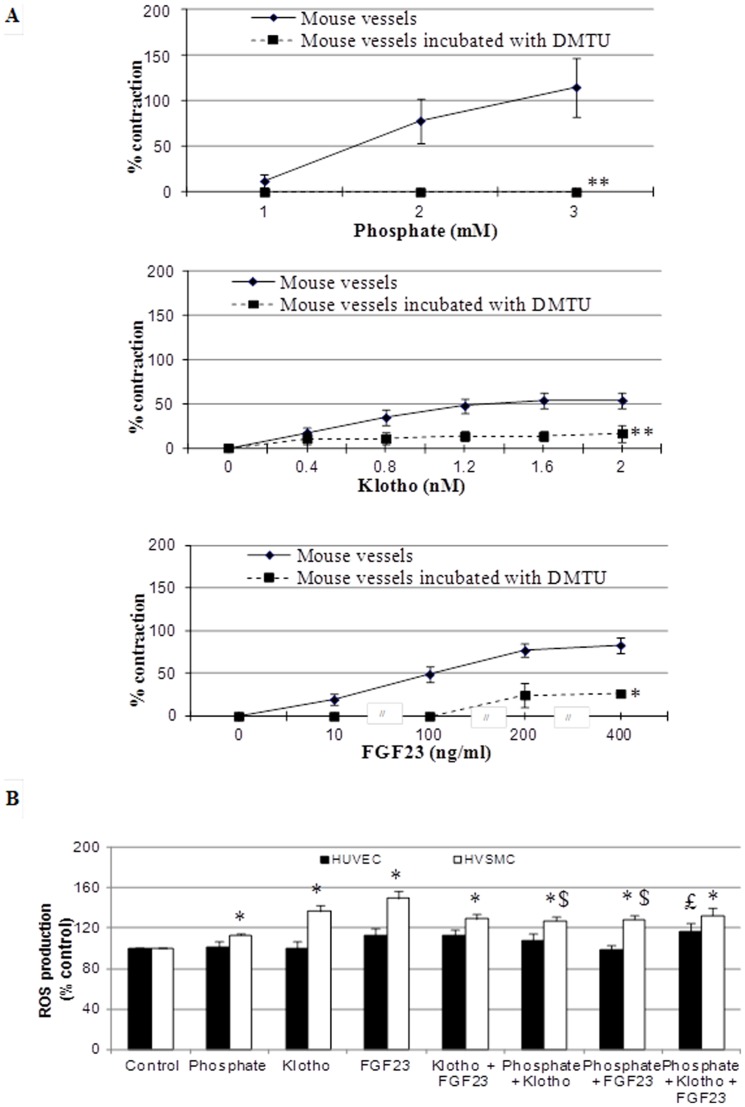
Direct effects of phosphate, Klotho and FGF23 on vascular reactivity and on H_2_O_2_ concentration. A.Vascular reactivity on aortic rings *ex vivo.* Direct effects of phosphate (1.0–3.0 mM), Klotho (0–2.0 nM) and FGF23 (0–400 ng/ml) on isolated aortic rings in absence or in the presence of ROS inhibitor, dimethylthiourea (10 mM). Contraction values are expressed as percentage of the contraction obtained with 70 mM KCl. * p<0.001, ** p<0.0001 vs. mouse vessels alone. N, 4 per experiment. B. Direct effects of phosphate (2.0 mM), Klotho (1.6 nM) and FGF23 (10 ng/ml), alone or associated, on H_2_O_2_ concentration in human umbilical vein endothelial cells (HUVECs) and human vascular smooth muscle cells (HVSMCs). * p<0.0005 vs. HVSMCs control group, $ p<0.005 vs HVSMCs phosphate group, £ p<0.05 vs. HUVECs control group. N, 7 per experiment.

As demonstrated previously [19], phosphate exhibited a vasoconstrictor effect on aorta rings in a dose-dependent manner and osmolarity kept constant even after addition of phosphate. Exposure of aorta rings to either Klotho or FGF23 induced concentration-dependent contractions within 1 min (**Figure 1A**). This effect was paralleled by an increase of H_2_O_2_ concentration in HVSMCs by phosphate (2.0 mM), Klotho (1.6 nM) and FGF23 (10 ng/ml) (**Figure 1** **B**). The contractions of aortic rings obtained with phosphate, Klotho or FGF23 were abolished by dimethylthiourea (**Figure 1A**). Consistent with the increase in ROS concentration in HVSMCs, ERK phosphorylation of mouse aorta increased in response to phosphate (163±39), Klotho (151±21) or FGF23 (287±119) respectively (p<0.05 vs control).

**Figure 2:**
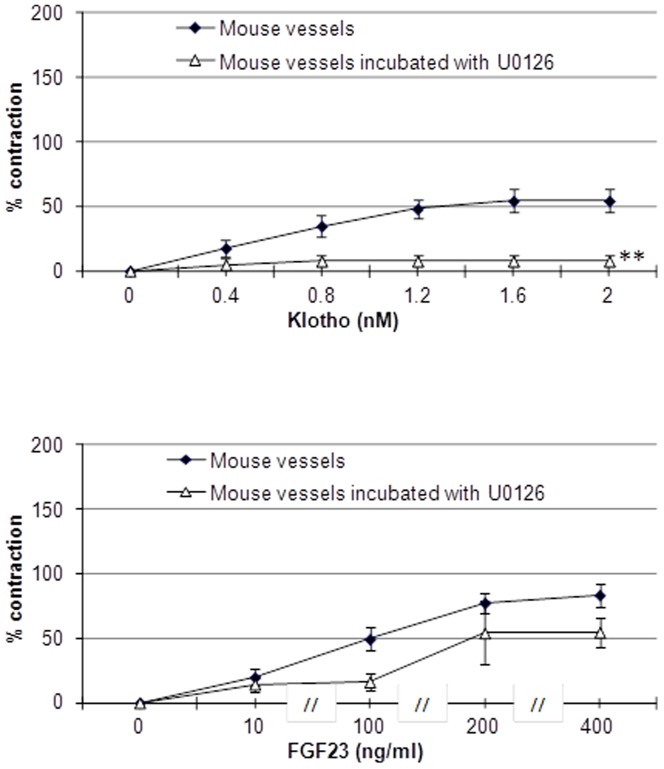
Vascular reactivity on aortic rings *ex vivo.* Direct effects of phosphate (1.0–3.0 mM), Klotho (0–2.0 nM) and FGF23 (0–400 ng/ml) on isolated aortic rings in absence or in the presence of ERK inhibitor, U0126 (10 μM). Contraction values are expressed as percentage of the contraction obtained with 70 mM KCl. ** p<0.0001 vs. mouse vessels alone. N, 4 per experiment.

The use of ERK inhibitor U0126 strongly reduced contraction obtained with Klotho. The effect of ERK inhibitor on contraction induced by FGF23 was much less marked (**Figure 2**).

### Attenuation of FGF23-induced vasoconstriction by Klotho, probably via enhanced NO generation

**Figure 3:**
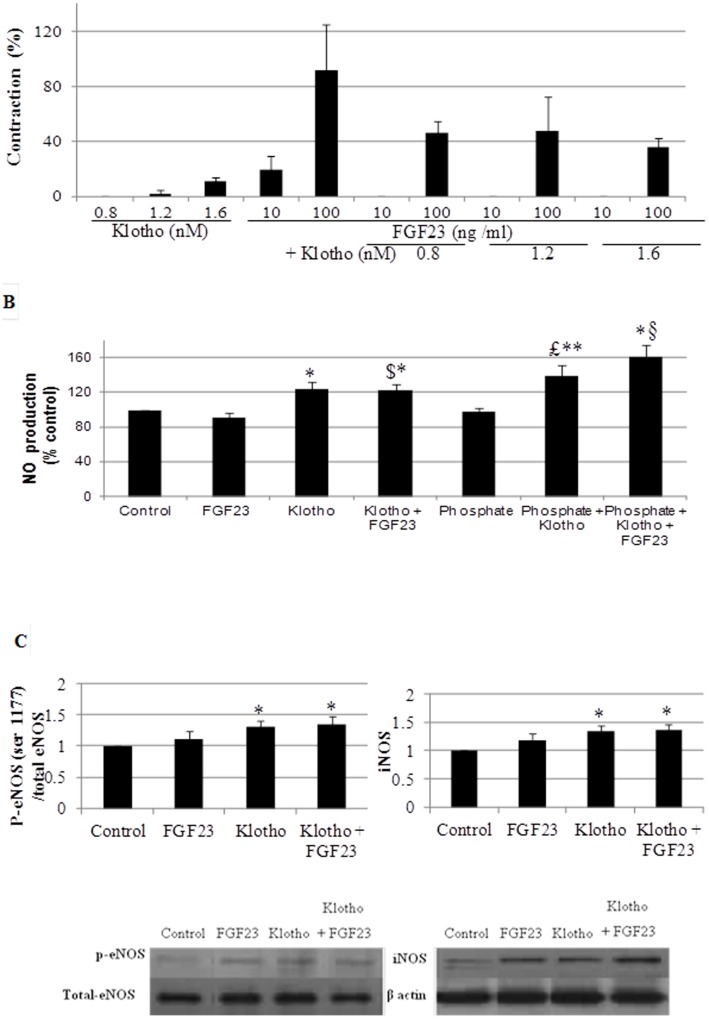
Effects of Klotho associated with FGF23 on vascular reactivity, NO production and eNOS and iNOS expressions. A.Vascular reactivity on aortic rings *ex vivo.* Vascular effects of FGF23 (10, 100 ng/ml) in association with Klotho (0.8, 1.2, 1.6 nM). Contraction values are expressed as percentage of the contraction obtained with 70 mM KCl. N, 4 per experiment. B. Effects of FGF23 (10 ng/ml), Klotho (1.6 nM) or phosphate (2.0 mM), alone or associated, on NO production, (N, 6 per experiment) in HUVECs. * p<0.05, ** p<0.005 vs. control group, $ p<0.05 vs FGF23 group, £ p<0.005 vs phosphate group, § p<0.05 vs. Klotho group. C. Effects of FGF23 (10 ng/ml), Klotho (1.6 nM), or the two combined on eNOS expression (N, 4–6 per experiment), and iNOS de novo expression (N, 4–6 per experiment) in HUVECs. * p<0.05 vs. control group.

In contrast to the vasoconstrictor effects of Klotho or FGF23 alone, the contractile response obtained with FGF23 at 10 ng/ml or 100 ng/ml, was completely abolished or significantly attenuated by pre-treatment with Klotho at 0.8, 1.2 or 1.6 nM (**Figure 3A**).

The increase in H_2_O_2_ concentration in HVSMCs by exposure to either Klotho or FGF23 remained unchanged by co-exposure to Klotho and FGF23 (**Figure 1B**). Co-exposure to Klotho and FGF23 led to ERK activation in mouse aorta (100±0 for control vs 294±77 for Klotho and FGF23, p<0.05).

Moreover, Klotho increased NO concentration in HUVECs whereas FGF23 did not. Co-exposure maintained Klotho effect (**Figure 3B**).

Exposure of HUVECs to Klotho alone or in association with FGF23 increased eNOS phosphorylation at serine 1177 (the active form of eNOS). Moreover, Klotho induced iNOS de novo expression, and this effect was maintained after co-exposure with FGF23 (**Figure 3C**).

### Induction of vascular relaxation by Klotho in phosphate-preconstricted aortic rings, probably via enhanced NO generation

**Figure 4:**
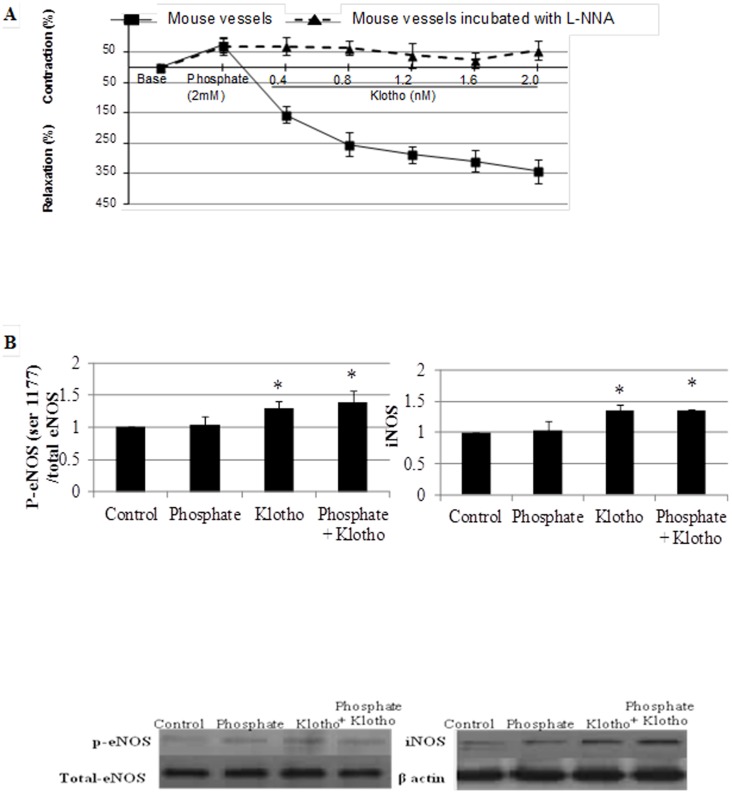
Effects of phosphate associated with Klotho on vascular reactivity and eNOS and iNOS expressions. A. Effects of phosphate (2.0 mM) combined with Klotho (0.4, 0.8, 1.2, 1.6, 2.0 nM) on aortic rings ex vivo. Contraction values are expressed as percentage of the contraction obtained with 70 mM KCl. Relaxation was calculated taking into account the maximal contraction obtained with phosphate 2.0 mM alone. The possible involvement of NO was studied by incubating aortic rings with L-NNA for 30 min before exposure to phosphate and Klotho. N, 4 per experiment. B. Effects of phosphate (2.0 mM), Klotho (1.6 nM) or the two combined on eNOS expression (N, 4–6 per experiment) and iNOS de novo expression (N, 4–6 per experiment). * p<0.05 vs. control group.

In contrast to the vasoconstrictor effect of Klotho alone, the addition of Klotho induced a concentration-dependent relaxation in phosphate preconstricted vessels (**Figure 4A**) or in phenylephrine preconstricted vessels (data not shown). Maximal Klotho induced vascular relaxation was obtained within 200 sec. This relaxant effect was abolished by endothelium removal (data not shown), indicating the involvement of endothelium-derived factors. Consistent with this hypothesis, the relaxation effect of Klotho on phosphate-preconstricted vessels was blocked by the addition of L-NNA, a competitive inhibitor of NOS with selectivity for the neuronal and endothelial isoforms of the enzyme (**Figure 4A**).

The addition of Klotho significantly augmented the concentration of H_2_O_2_ induced by phosphate alone only in HVSMCs (**Figure 1B**). Klotho and phosphate alone or in combination induced ERK activation in mouse aortas (100±0 for control vs 261±53 for Klotho and phosphate, p<0.05).

Phosphate induced a significant increase in NO concentration and activated eNOS only in presence of Klotho. Moreover, Klotho alone was able to induce iNOS de novo expression and this effect was maintained even in presence of phosphate (**Figures** **3B** **and** **4B**).

### Enhancement of vascular contraction by FGF23 in phosphate-preconstricted vessels, possibly by an induction of ROS expression

**Figure 5:**
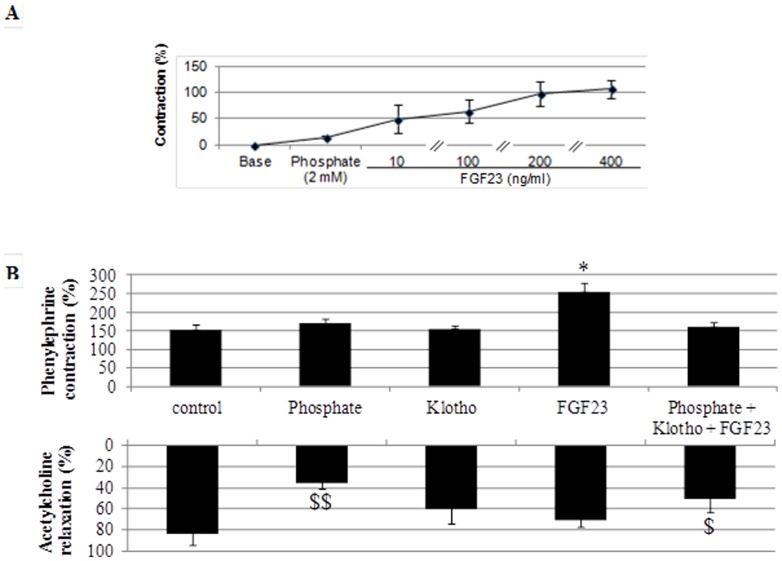
Vascular effects of phosphate (2.0 mM) associated with FGF23 (10–400 ng/ml) (A) and contraction obtained with phenylephrine and relaxation obtained with acetylcholine after incubation of aortic rings in presence of phosphate (2.0 mM), Klotho (1.6 nM), FGF23 (10 ng/ml) or the three combined (B). * p<0.005 vs. all other groups, $ p<0.05 or $$ p<0.001 vs control group.

The addition of FGF23 induced a further, dose-dependent contraction in phosphate preconstricted vessels. Although FGF23 alone was able to induce concentration-dependent contractions this effect was thus enhanced when FGF23 was associated with phosphate (**Figure 5A**). The addition of FGF23 significantly augmented H_2_O_2_ concentration induced by phosphate alone only in HVSMCs (**Figure 1B**). FGF23 and phosphate alone or in combination induced ERK activation in mouse aortas (100±0 for control vs 290±84 for phosphate and FGF23, p<0.05).

### Reduction of endothelium dependent vascular relaxation by Klotho in combination with FGF23 and phosphate

Although phosphate, Klotho, or FGF23 alone exhibited vasoconstrictor effect in mouse aorta rings no contractile response was observed upon co-incubation. The response to phenylephrine was increased by FGF23 and endothelium dependent relaxation decreased by phosphate. Co-incubation with the three compounds decreased endothelium dependent vasorelaxation induced by acetylcholine (**Figure 5B**).

Klotho, phosphate and FGF23 together increased H_2_O_2_ concentration both in HVSMCs and HUVECs (**Figure 1B**). This combination increased ERK activation in aortas (100±0 for control vs 320±119 for Klotho and phosphate and FGF23, p<0.05).

The three compounds together increased NO concentration in HUVECs via an increase of eNOS expression and induction of de novo iNOS expression (p<0.05 vs control, **Figure 3B**).

## Discussion

In the present acute experiments, we demonstrate for the first time that both FGF23 and Klotho directly stimulate arterial vasoconstriction in a concentration-dependent fashion. These actions were found to occur independently of each other. However, their combined effects on the vessel wall are more complex, owing to apparently opposing actions in terms of local oxidant generation and NO production.

In addition to its major expression in kidney, parathyroid, and choroid plexus Klotho is expressed in human vascular tissue [7]. Both Klotho and FGF23 participate in the regulation of vascular tone [16], [17]. Moreover, Klotho overexpression improves endothelial function through increased NO production [18].

In the present study we show that Klotho induced a direct dose dependent vasoconstriction. In a previous study using same experimental model, we observed a direct, acute vasoconstrictive effect of phosphate which was associated with enhanced ROS production [19]. According to previous studies done in our *in vitro* HUVEC model Klotho was unable to induce H_2_O_2_ production [20]–[22]. In contrast, in the present *in vitro* HVSMC model addition of Klotho induced an excessive H_2_O_2_ concentration. When using cumulative concentrations of Klotho, the vasoconstriction obtained for each concentration was increased compared to a single concentration (**Figures 1A** **,** **3A**). This led us to suppose that a relaxation factor was stimulated by Klotho as well. This hypothesis was reinforced by the observation of a relaxation effect of Klotho on phosphate preconstricted aorta. Under the same condition, Klotho's relaxant effect was abolished by endothelium removal, indicating the involvement of endothelium-derived factors. Consistent with this hypothesis, the relaxation effect of Klotho was blocked by the addition of L-NNA, a competitive inhibitor of NOS. Moreover, Klotho was able to increase endothelial NO concentration. These results are in accordance with previous studies demonstrating a decrease of endothelium-dependent relaxation, NO metabolites such NO_2_^−^ and NO_3_^−^ and NO in aortas of heterozygote Klotho mice, and a total lack of relaxation in aortas of Klotho deficient mice [17], [23]. Our study shows that Klotho can directly induce eNOS phosphorylation and iNOS expression, as based on L-NNA experiments which inhibit both enzymes, leading to increased NO concentration and relaxation of preconstricted aorta. Klotho induced iNOS expression suggesting that it may stimulate inflammatory responses as suggested by other authors [26]. Klotho kept its eNOS phosphorylation capacity even in presence of phosphate and thereby reverted its vasoconstrictor effect. A beneficial vascular role of Klotho has also been demonstrated in the context of hyperphosphatemia. The Klotho concentrations used in the present study were comparable to those used by Hu et al. [24].

Thus the vascular action of Klotho appears to reflect a balance between its effects on ROS and NO concentration. In single Klotho exposure experiments the decrease of vasoconstriction appeared to be mainly linked to NO stimulation. In contrast, incubation with increasing Klotho concentrations induced vasoconstriction, probably due to NO degradation by ROS and the longer half-life of ROS compared to NO. A subtle balance between NO and ROS effects could be one of the explanations for the present controversy on the vascular effects of Klotho.

A previous report showed a positive association between high FGF23 serum concentrations and vascular dysfunction [16]. In line with this finding, we show for the first time a direct, acute vascular effect of FGF23 at serum concentrations observed in uremic patients [25]. As previously suspected, but not proven so far, FGF23 alone exerted a marked vasoconstrictor effect in aortic rings. In contrast, Lindberg et al failed to observe an FGF23 effect on aorta reactivity [27]. The discrepancy could be explained by their use of small resistance vessels and lower FGF23 concentrations. In our study, the FGF23 vasoconstrictor effect was found to be related to ROS concentration in HVSMC and associated with ERK1/2 activation in aortic tissue. The activation of ROS appeared to be the only contribution of FGF23 to vascular dysfunction. In contrast to Klotho, the vasoconstrictive effect in response to increasing concentrations of FGF23 was similar to that obtained with a unique concentration and there was no effect on NO. Taken together, our findings are compatible with the hypothesis that in vascular tissues, FGF23 exerts Klotho-independent effects, and that the mechanism of action of FGF23 may differ from one type of tissue to another. The importance of the cardiovascular effects of FGF23 excess and their independence of the Klotho coreceptor have recently been questioned in a study done in patients with a broad range of kidney function impairment [28].

Strikingly, the exposure of phosphate pre-contracted vessels to increasing levels of FGF23 further increased aortic constriction and ROS concentration, suggesting a synergistic effect. Thus in contrast to Klotho, FGF23 was unable to reverse the contractile effects of phosphate. Moreover, FGF23 at physiological concentration (10 ng/ml) exerted vasoconsctrictive, Klotho independent vascular effects.

When Klotho was associated with FGF23, the vasoconstriction induced by FGF23 could be abolished by Klotho, probably via enhanced NO production. Interestingly, Klotho also mitigated the direct effects of combined phosphate and FGF23 on aortic contractility, thereby protecting the vessel wall. Similar complex interactions between Klotho and FGF23 at the site of the VSMC have recently been observed by Lim et al, although they examined longer time periods [7]. In the setting of CKD, ROS excess may trigger signalling events that further exacerbate VSMC proliferation and vascular remodelling. However, in physiological condition ROS such as H2O2 play an essential role in signal transduction and regulation of vascular function.

Finally, when studying the combined effects of phosphate, Klotho and FGF23, we observed no direct change in vascular function despite the vasoconstriction induced by each substance separately.

After incubation in presence of phosphate or Klotho or FGF23, the response to phenylephrine was increased by FGF23 and endothelium dependent relaxation was decreased by phosphate. The combined stimulation with phosphate and Klotho and FGF23 did not change the contraction induced by phenylephrine, but this combination decreased the endothelium dependent vasorelaxation induced by acetylcholine

Moreover, the combined exposure of these three compounds led to enhanced ROS concentration by HUVECs. As to eNOS activation, it was comparable when HUVEC were exposed to Klotho alone or in association with phosphate and/or FGF23. Thus with triple exposure there is an imbalance between the contraction effect mediated by smooth muscle generated ROS and the relaxation effect mediated by endothelial NO. The predominant phenomenon appears to be endothelial ROS generation opposing the endothelial NO effect. This observation is in accordance with the partial inhibition of endothelium dependent relaxation in response to acetylcholine.

The present study has some limitations. We did not test the different compounds in vivo. Moreover, in contrast to the study of Faul et al [15], we did not explore the role of FGF receptors in the observed FGF23 effects. In a subsequent study, it will be interesting to test the effect of FGF receptor inhibitors on arterial contraction induced by FGF23.

In conclusion, although separate in vitro exposure to phosphate, soluble Klotho, or FGF23 stimulates aorta contraction directly, Klotho mitigates the vasoconstrictive effects of phosphate and FGF23 and thereby protects the vessel wall against potentially excessive vasoconstrictive effects of high phosphate and FGF23 concentrations. Our findings support the theory that Klotho deficiency in CKD is noxious whereas Klotho sufficiency is protective against the negative effects of high phosphate and FGF23 concentrations, which are additive. Since both Klotho deficiency and FGF23 excess appear to be deleterious one could speculate about the relative merits of potential therapeutic effects of Klotho stimulation versus FGF23 suppression. Since high phosphate, Klotho and FGF23 concentrations combined can induce endothelial cell dysfunction the correction of Klotho deficiency alone may not be sufficient.
